# Surfactant Self-Assembling and Critical Micelle Concentration: One Approach Fits All?

**DOI:** 10.1021/acs.langmuir.0c00420

**Published:** 2020-05-06

**Authors:** Diego Romano Perinelli, Marco Cespi, Nicola Lorusso, Giovanni Filippo Palmieri, Giulia Bonacucina, Paolo Blasi

**Affiliations:** School of Pharmacy, University of Camerino, 62032 Camerino, Italy

## Abstract

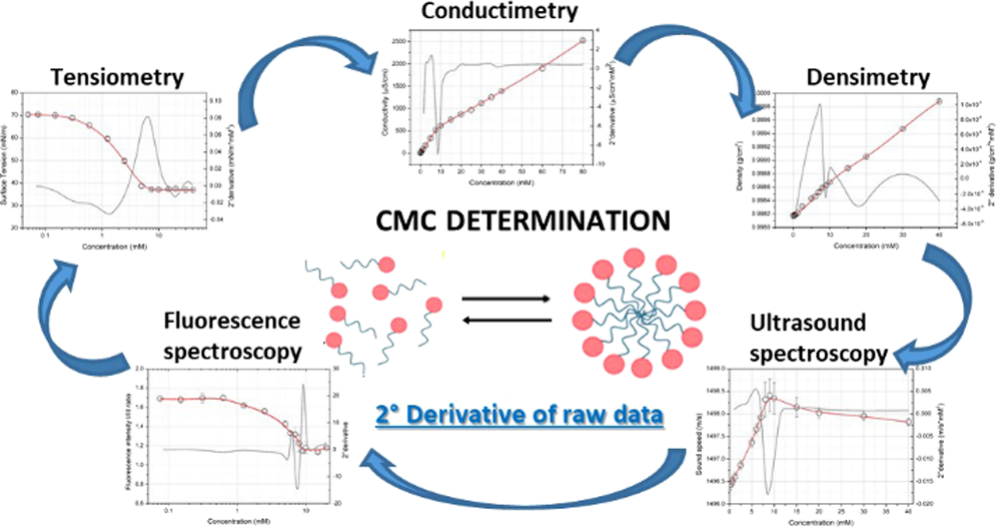

Critical micelle concentration (CMC) is the main chemical–physical parameter to be determined for pure surfactants for their characterization in terms of surface activity and self-assembled aggregation. The CMC values can be calculated from different techniques (*e.g*., tensiometry, conductivity, fluorescence spectroscopy), able to follow the variation of a physical property with surfactant concentrations. Different mathematical approaches have been applied for the determination of CMC values from the raw experimental data. Most of them are independent of the operator, despite not all of the fitting procedures employed so far can be applied in all techniques. In this experimental work, the second derivative of the experimental data has been proposed as a unique approach to determine the CMC values from different techniques (tensiometry, conductimetry, densimetry, spectrofluorimetry, and high-resolution ultrasound spectroscopy). To this end, the CMC values of five different surfactants, specifically three anionic (sodium dodecyl sulfate, sodium deoxycolate, and *N*-lauroyl sarcosinate) and two nonionic, such as polyethylene glycol ester surfactants [polyethylenglicol (8) monostearate and polyethylenglicol (8) monolaurate], have been determined by this approach. The “second-derivate” approach provides a reliable determination of the CMC values among all of the techniques investigated, which were comparable to those calculated by the other operator-free routinely methods employed, such as segmental linear regression or Boltzmann regression. This study also highlighted the strengths and shortcomings of each technique over the others, providing an overview of the CMC values of commonly used anionic and nonionic surfactants in the pharmaceutical field, determined by employing different experimental approaches.

## Introduction

Surfactants are amphiphilic molecules, composed of a hydrophobic and hydrophilic portion, of large use in different technological fields and industrial applications.^1^ The global surfactant market has been estimated as $43 655 million in 2017 and is expecting to reach $66 408 million by 2025, with a compound annual growth rate (CAGR) of 5.4%.^2^ The growth and high demand for surfactants account for their wide usage ranging from household detergents and personal care products to industrial applications as cleaners, food, textiles, plastics processing aids, or oilfield and agricultural chemicals. As regards pharmaceutical and cosmetic formulations, surfactants are excipients required for the stabilization of all dispersed systems. Specifically, they act as emulsifiers in the formulation of emulsions and creams and as stabilizers,^3,4^ flocculating, or wetting agents in the formulations of suspensions. Moreover, many surfactants (*e.g*., sodium dodecyl sulfate (SDS), polysorbates) are employed to increase the apparent solubility of poorly soluble drugs in an aqueous environment, acting as solubilizing agents.^5^ These molecules are also able to interact with biological membranes, thanks to their amphiphilic structure, thereby increasing drug permeability across skin or mucosa.^6^ As such, several studies have been conducted to evaluate the potential use of different classes of amphiphiles as drug permeability enhancers.^7,8^ All of these interesting and exploitable applications come from the amphiphilic structure of surfactants, which determines their chemical–physical properties. In fact, being amphiphilic, they are able to be adsorbed at the interface, decreasing the Gibbs free energy of the two-phases systems, thereby exerting a stabilizing effect. In addition, surfactants show also self-assembling properties as a function of concentration and, to a less extent, temperature. Once all surfaces are saturated, surfactants start to self-assemble in water into supramolecular aggregates, whose structure is determined by the geometric factor referred to as the “critical packing parameter”. These structures for surfactants are generally called micelles, indicating supramolecular aggregates, in which the packing of the hydrophobic tails forms the core, while the hydrophilic heads are exposed outside in contact with the aqueous environment.^9,10^ The minimum concentration of surfactant at which micelles form is termed as “critical micelle concentration” (CMC) and represents one the most important chemical–physical parameters to be determined for these amphiphilic molecules. The properties of surfactants and, therefore, their applications are strongly influenced by the physical state of surfactants as unimers or micelles. For instance, the solubilizing effect appears only at concentrations much above CMC since, in most cases, it is proportional to the number of micelles in water.^5^ The toxicity of surfactants is, also, dependent on CMC since toxic effects, especially for noncharged surfactants, appear at concentrations close to or higher than CMC.^11−13^ CMC of surfactants mainly depends on the hydrophobicity of the amphiphiles (*e.g*., length of the hydrophobic tail) and is strongly influenced by the characteristics of solutions (*e.g*., presence of salts). CMC can be determined using several experimental approaches, which could be grouped into tensiometric (*e.g*., force or optical tensiometry), electrochemical (*e.g*., conductimetry), optical (dynamic light scattering), or spectroscopic (*e.g*., fluorescence or ultrasonic spectroscopy) techniques.^14−16^ Actually, any technique able to detect a marked variation in the measured parameter related to the chemical–physical properties below and above CMC and, specifically, to the unimeric or micellar state of surfactants, can be employed. Despite different techniques generally provide quite comparable CMC values among the tested surfactants, it is not unequivocal and straightforward how to treat the experimental data to calculate the CMC values. Actually, different mathematical approaches have been applied to calculate CMC from the experimental data obtained from different techniques (*e.g*., a linear regression model for tensiometry, a nonlinear regression model for spectrofluorimetry).^17−19^ In addition, different approaches have been proposed for the same technique, showing sometimes only partially advantages over each other.^20,21^ We proposed here the use of a single approach, as the second derivative of the experimental data from different techniques (tensiometry, conductimetry, densimetry, spectrofluorimetry, and high-resolution ultrasound spectroscopy) to determine the CMC values. As such, five different surfactants were chosen as a model, specifically, three anionic (sodium dodecyl sulfate, sodium deoxycolate, and *N*-lauroyl sarcosinate) and two nonionic, such as polyethylene glycol (PEG) ester surfactants (polyethylenglicol (8) monostearate and polyethylenglicol (8) monolaurate). The strengths and concerns of using a technique over the others for the different surfactants have been highlighted, and the obtained CMC values have been compared.

## Experimental Section

### Materials

Sodium dodecyl sulfate (SDS, purity ≥98.5%; CMC 7–10 mM, according to the manufacturer), deoxycholic acid, sodium salt monohydrate (NaDC, purity >98%; CMC 2–6 mM, according to the manufacturer), and *N*-lauroyl sarcosine sodium salt (SDDS, purity >98.5%; CMC 14.6 mM, according to the manufacturer) were purchased from Sigma-Aldrich (St. Louis, MO). Polyethylenglicol (8) monostearate (PEG8-S, Cithrol 4MS) and polyethylenglicol (8) monolaurate (PEG8-L, Cithrol 4ML) were obtained from Croda (Goole, U.K.). All surfactants were used as received without further purification. Ultrapure water was produced using a laboratory deionizer (Osmo lab UPW2, γ 3; Castelverde, Italy).

### Tensiometric Analysis

Different concentrations of surfactants were prepared in ultrapure water and analyzed at 25 °C using a tensiometer “DCA-100 (First Ten Angstroms)” according to the “Du Noüy ring” method. Every reported surface tension value was the average of three consecutive measurements. Data were the mean ± standard deviation of three independents measurements.

### Conductometric Analysis

The specific conductivity (μS/cm) of surfactant solutions (SDS, NaDC, SDDS) in water was measured at 25 °C using a MicroCM 2200 conductimeter (Crison, Spain). All concentrations were measured three times. Data were the mean ± standard deviation of three independents measurements.

### Fluorimetric Analysis

Three microliters of pyrene solution in methanol at a concentration of 2 μM were added to the aqueous surfactant solutions. Pyrene spectrum (excitation wavelength 334 nm) was recorded in the range between 200 and 700 nm with a 2.5 nm excitation slit and a 2.5 nm emission slit. Each recorded spectrum was the sum of ten acquisition. Analyses were performed at 25 °C using an LS 55 fluorescence spectrometer (PerkinElmer) equipped with a thermostatic bath (HAAKE C25P). The ratio of peak intensity I (λ = 372 nm) to peak intensity III (λ = 384 nm) of the emission spectrum of pyrene was plotted against surfactant concentrations. Data were the mean ± standard deviation of three independent measurements.

### Densimetric Analysis

The density of surfactant solutions of different concentrations in water was measured by a DMA 5000 M high-resolution densimeter (Anton Paar, Graz, Austria) at 25 °C using an oscillating U-tube method. Each solution was analyzed three times. Data were the mean ± standard deviation of three independents measurements.

### Ultrasound Spectroscopy

Ultrasound parameters such as sound speed (m/s) and attenuation (1/m) were recorded for each surfactant concentration in water through a high-resolution ultrasound spectrometer (HR-US 102; Ultrasonic Scientific, Ireland) at 25 °C. The instrument is fitted with two cells, one filled with 1 mL of sample and the other one with 1 mL of water as a reference. The absolute sound speed and attenuation were measured for 300 s for each surfactant concentration. Data were the mean ± standard deviation of three independents measurements.

### Data Analysis and CMC Determination

Data points from each technique were interpolated by a nonuniform rational basis spline (NURBS) algorithm using TableCurve 2D software. Then, the second derivative of the interpolated curve was calculated. The CMC values were obtained from the maximum point individuated by the second derivative of each surfactant *vs* concentration plot.

As a reference, the CMC values were also calculated by the segmental linear regression of raw data (GraphPad Prism 6 software) and by the Boltzmann nonlinear regression for fluorescence raw data according to the following equation
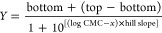
1where top and bottom are the plateaux of the curve in the unit of *Y*-axis and hill slope is the steepness of the curve, and the CMC value was calculated from the center of the sigmoid.

## Results and Discussion

### Tensiometric Measurements

**Figure 1:**
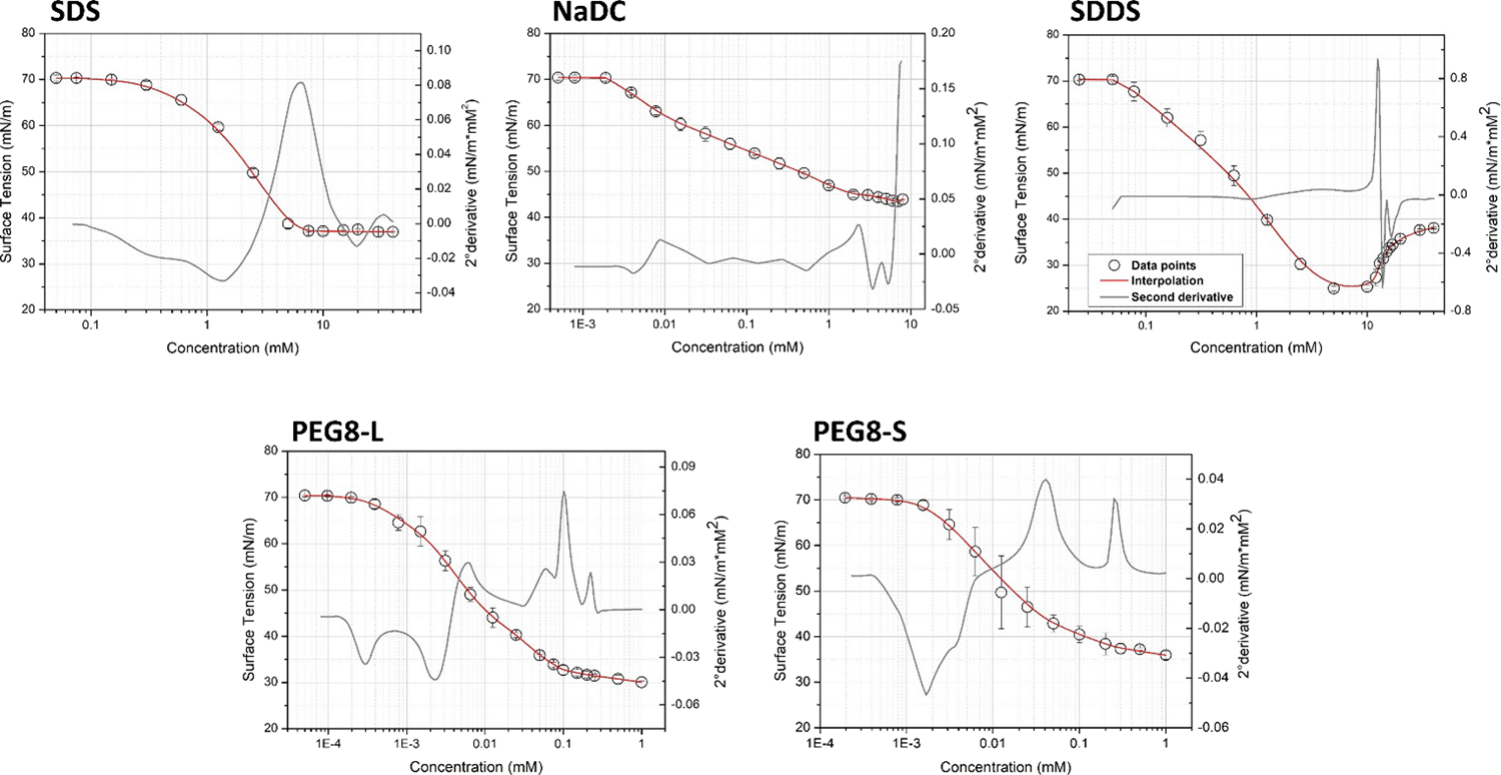
Surface tension *vs* concentration for ionic (SDS, NaDC, SDDS) and nonionic (PEG8-L and PEG8-S) surfactants.

The surface tension *vs* concentration plots for all analyzed surfactants are shown in Figure 1. The profile for SDS is that typical for a pure surfactant since two plateaux are linked by a region in which the surface tension decreases as a function of concentration. The two plateaux correspond to the range of surfactant concentration not affecting the surface tension. In the first plateau, surface tension is close to that of pure water (72 mN/m) since the low surfactant concentration does not affect the surface tension. The second plateau is related to the surfactant concentration, above CMC, in which the air–water surface is saturated by surfactant molecules. The neat change in the slope of surface tension raw data can be used for the calculation of CMC.^22^ In the case of NaDC, not a clear plateau was observed in the range of concentrations (2–6 mM) reported by the manufacturer as the CMC range. This can be explained by the stepwise aggregation of NaDC.^23^ According to the model of Small, first, the so-called “primary micelles” form due to the interactions between the hydrophobic portions of NaDC. Subsequently, these micelles self-assemble into larger aggregates known as “secondary micelles”.^24^ In this way, more complicated equilibria occur between the unimeric and micellar states of the amphiphile, in the case of NaDC with respect to classical surfactants, which account for the reported CMC as a range of concentrations instead of a single value. The surface tension values recorded for SDDS show a different profile, which may be ascribed to that of a surfactant containing a small amount of impurities, as reported in the literature.^25^ In this case, the surface tension decreases down a minimum and then increases up again to a plateau. The initial point of the plateau is generally considered the CMC of the surfactant.^26^ The observed surface tension *vs* concentration profile for SDDS has been already reported in the literature, in most of the cases without any detailed reference to the purity of the amphiphile.^26−28^ To assess the eventual presence of impurities, electrospray ionization (ESI) mass spectrometry was performed on all surfactants (Figures S1–S7). The mass spectra of SDDS clearly shows a molecular ion at 270.2 *m*/*z*, which is related to the amphiphilic compound, and a signal with a very low intensity at 199.2 *m*/*z*, which could be ascribed to the presence of a small amount of lauric acid (MW, 200.3) as an impurity (Figure S3). As regards nonionic PEGylated surfactants (PEG8-L and PEG8-S), the saturation of the air–water surface by the amphiphile, as indicated by a plateau in the surface tension values, was reached at concentrations lower than those for anionic surfactants (generally below 1 mM). Particularly, one main maximum value in the second derivative plot was individuated around the concentration of 0.1 mM and recognized as the CMC for PEG8-L. On the contrary, two main maximum values were individuated for PEG8-S in the second derivative plot: one around 0.04 mM and the other around 0.2 mM, leading to uncertainty in the determination of CMC. To investigate the purity and actual composition of the commercial nonionic surfactant formulations (PEG8-L and PEG8-S), mass analysis was performed (Figures S4–S7). Actually, negative mode ESI mass spectra revealed the presence of hydrophobic chains linked by ester bondage to PEG of different lengths, whose C12 chain and C18 chain were prevalent for PEG8-L and PEG8-S surfactants, respectively. Traces of compounds with the shortest acyl chain (C10–C16) as impurities in the PEG8-S surfactant have been also revealed by differential scanning calorimetry (DSC) analysis. The thermogram of PEG8-S clearly shows, in addition to the melting of this compounds (around 30–35 °C), other endothermic events at lower temperatures (from −10 to 10 °C), which can be attributed to PEG esters with the shortest acyl chains (Figure S8).

### Conductivity Measurements

**Figure 2:**
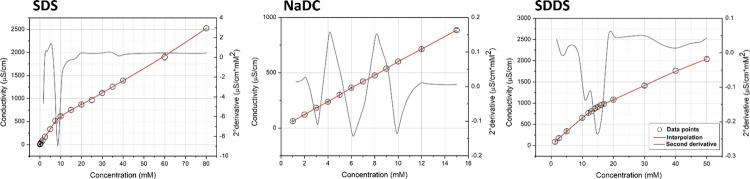
Specific conductivity *vs* concentration plots for ionic (SDS, NaDC, SDDS) surfactants.

Conductivity is a common technique to determine CMC for ionic surfactants, which behave as electrolytes in water. This technique cannot be used for nonionic surfactants (PEG8-L and PEG8-S) since these surfactants have a negligible effect on the conductivity of the solution. For SDS and SDDS, two linear segments with different slopes can be recognized and ascribed to the unimeric and micellar states of the surfactant in solution. Specifically, the increase of conductivity per unit of concentration is higher when the surfactants in solution are present as unimers with respect to the presence of micelles.^29^ CMC can be calculated from the breakpoint of raw data, which can be clearly identified for SDS and SDDS (Figure 2). Contrarily, the change in the slope of raw data is not evident for NaDC, resulting in a not marked variation in mobility after the aggregation of unimers into micelles (Figure 2). This can be explained by the low aggregation number of NaDC micelles and, consequently, the negligible effect of the inclusion of counterions within the micelles.^30,31^

### Density Measurements

**Figure 3:**
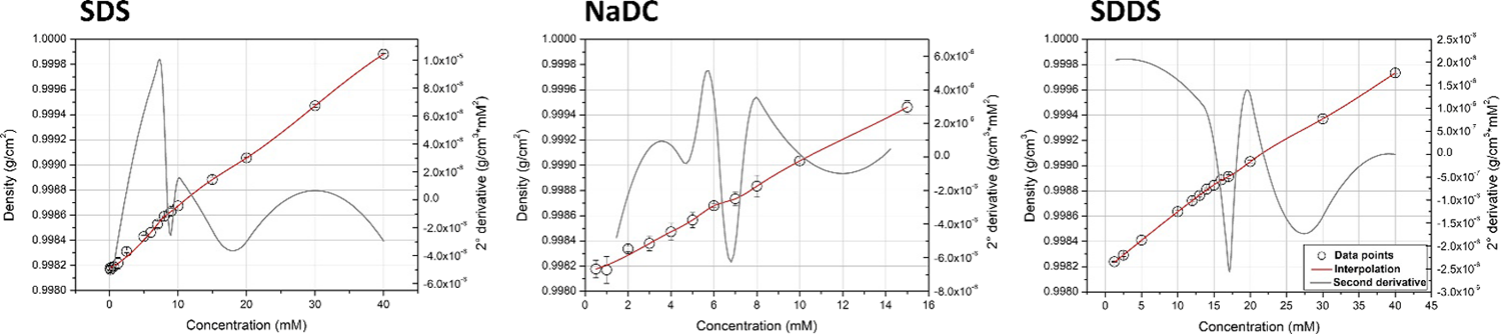
Density *vs* concentration plots for ionic (SDS, NaDC, SDDS) surfactants.

Density is another physical parameter of solutions, which changes as a function of the aggregation state of surfactants.^32^ Specifically, the increase of density of a solution at the unimer state per unit mass of the surfactant is higher than its increase at a surfactant concentration in which micelles are present. This is related to the different volume fractions in the solution of unimers and micelles. The volume fraction of unimers is higher than that of micelles because of their higher hydration. Consequently, water is more bounded in the presence of unimers with respect to micelles. Micellization, indeed, is a dehydration process, leading to a large increase in the free water with respect to bounded water. Therefore, the increase of volume per unit mass of the surfactant as unimers is lower than that as micelles. For all analyzed surfactants, CMC appears as a stepwise deflection in the increase of density over concentration (Figure 3), and it can be clearly identified as the maximum of the second-derivative trace. Moreover, the extent of increase of density in the unimeric state (slope) is dependent on the different grade of hydration and the molecular weight of the surfactant. The slope (8.9 × 10^–5^ μS/cm mM), in fact, is markedly higher for NaDC with respect to the other two surfactants, as a function of their molecular weights (MW of NaDC is higher than MW of SDS and SDDS). Moreover, SDS and SDDS showed comparable slopes (5 × 10^–5^ and 4.5 × 10^–5^ μS/cm mM, respectively) due to their similar molecular weights (288 and 293 kDa, respectively) and the possible negligible differences in hydration of unimers, being both linear anionic surfactants with a 12-carbon hydrophobic tail (Figure S9). The density measurements cannot be reliably applied for the determination of CMC for nonionic surfactants since the variation in the density at very low surfactant concentrations was not appreciable and practically comparable to that of pure water, by considering the error associated with the measurement.

### Fluorescence Spectroscopy Measurements

**Figure 4:**
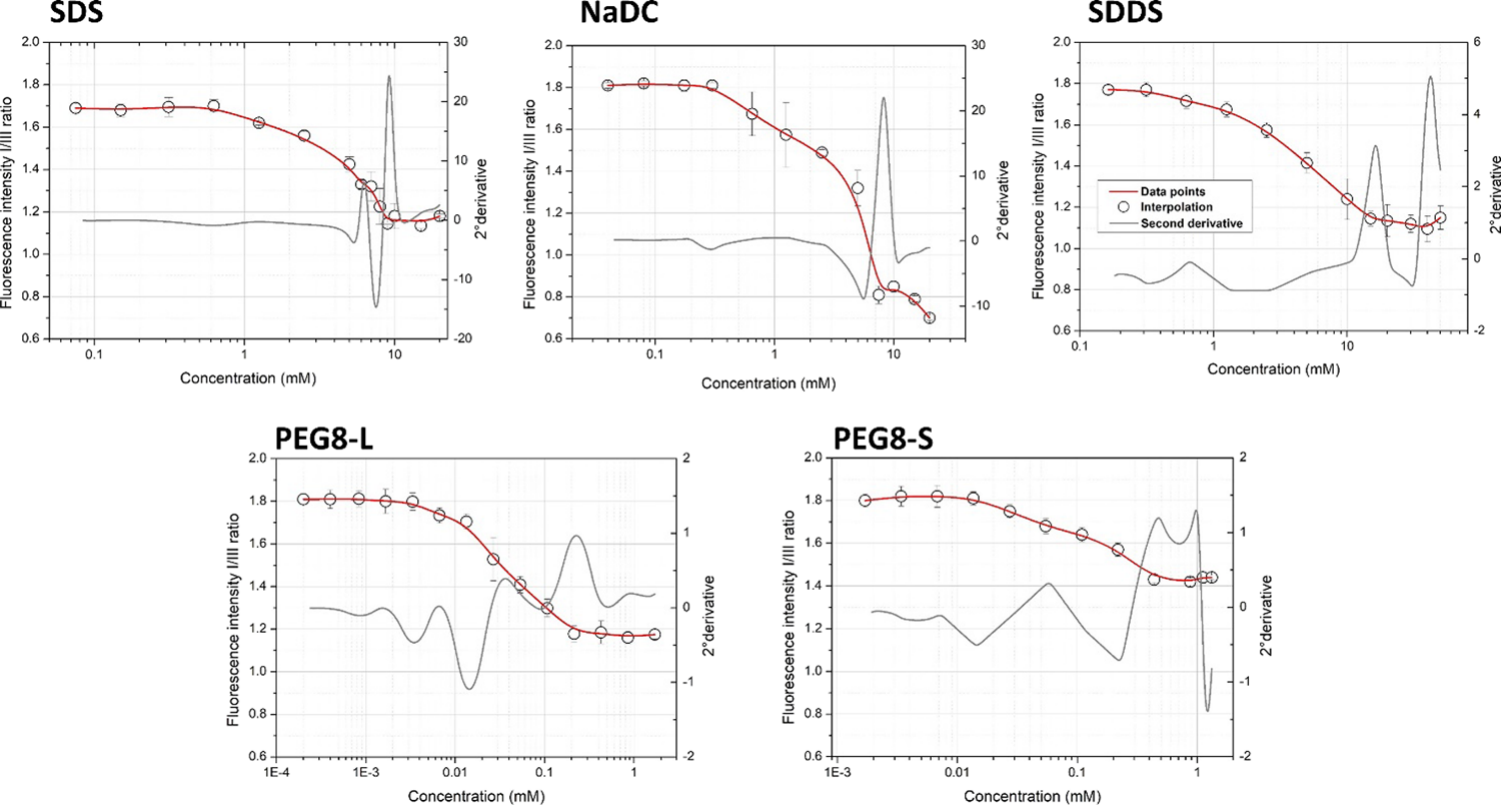
Fluorescence intensity (peak I/III) *vs* concentration plots for ionic (SDS, NaDC, SDD) and nonionic (PEG8-L and PEG8-S) surfactants.

The decrease in the fluorescence emission from I/III pyrene peaks over concentrations for all analyzed surfactants is shown in Figure 4. Such a decrease indicates that the microenvironment around pyrene (used as a fluorescent probe) changes with surfactant concentrations becoming more hydrophobic, as a consequence of pyrene interactions with the surfactant micelles. The profiles for SDS, SDDS, and PEG8-L have a well-shaped sigmoid, as already reported in the literature.^28,33^ On the contrary, two or more inflections in the decrease of I/III pyrene fluorescence emission are recognized for the polyethylenglicol ester surfactant PEG8-S^34,35^ and bile salts (as NaDC).^23^ These inflections are particularly evident for NaDC, reflecting the stepwise aggregation behavior of this amphiphile.^23^

### HR-US Measurements

High-resolution ultrasound spectroscopy is a powerful analytical tool for the characterization of the self-assembling behavior of amphiphilic compounds, including surfactants. This spectroscopic technique employs high-intensity ultrasounds at a low frequency (20–100 kHz) to study the structural properties of materials as a function of concentration or temperature in a fast, nondestructive, and reliable manner. Particularly, HR-US measures how the properties of the ultrasound wave change after traveling through the materials. Ultrasound waves, in fact, lose part of their energy and change the velocity of propagation as a function of the structure of the materials, resulting in a variation in the measured ultrasound parameters: sound speed and attenuation.

Sound speed represents the velocity of propagation of the ultrasound waves in the material and depends on the elasticity and density of the medium as expressed by the Laplace equation
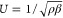
2where ρ is the density and β is the compressibility of the medium, which is defined as the relative change of the medium volume per unit of pressure applied by the ultrasonic wave.

Ultrasonic attenuation, instead, is referred to the decrease in the fluctuation amplitude consequent to the loss of energy occurring when the ultrasound wave travels through the material. Actually, any discontinuity inside the materials, including the formation of micelles, determines an increase of ultrasound attenuation.

**Figure 5:**
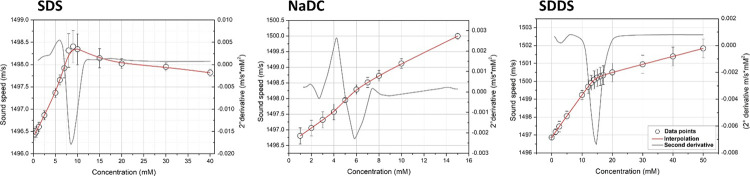
Sound speed *vs* concentration plots for anionic (SDS, NaDC, SDDS) surfactants.

As observed in Figure 5, sound speed generally increases with surfactant concentration. However, this increment is not linear but dependent on the aggregation state of surfactants (as unimers or micelles). Definitely, the sound speed profiles for surfactants are strongly determined by the CMC values. Specifically, below CMC, the sound speed is only affected by the properties of the amphiphiles in the unimeric state according to the following equation
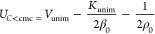
3where *V*_unim_ and *K*_unim_ are respectively the specific volume and the compressibility of surfactants in the unimeric state and β_0_ is the coefficient of adiabatic compressibility.

Above CMC, instead, sound speed is affected by both surfactants in the unimeric state (whose concentration is equal to CMC) and surfactants in the micellar states according to the following equation
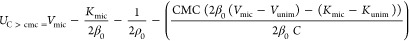
4where *V*_mic_ and *K*_mic_ are respectively the specific volume and the compressibility of surfactants in the micellar state.

As a consequence, below CMC, the increase in sound speed is linear and dependent mainly on surfactant concentration. Above CMC, the increase in sound speed is slower due to an increase in compressibility of the systems as a result of the loss of water bounded to the amphiphiles occurring during the micellization process. The presence of the terms *V*_mic_ and *K*_mic_ in the equation suggests also that the variation in sound speed above CMC is also dependent on the intrinsic structure of the micellar aggregates.

Some differences can be noticed in the sound speed profiles of the analyzed anionic surfactants (Figure 5). Particularly, the differences are clearly visible at concentrations above CMC since below the CMC all three surfactants showed the same increment in sound speed as revealed by the slope values (0.21, 0.28, and 0.23 m/s mM for SDS, NaDC, and SDDS, respectively). A remarkable change in the slopes was observed for SDS and SDDS. Indeed, while for SDDS, sound speed still increases above CMC (despite at a lower rate than below CMC), in the case of SDS, sound speed slightly decreases, probably reflecting the different hydration state of the micelles formed by the two surfactants. The formation of micelles for SDS could require more pronounced dehydration than SDDS, thereby increasing the amount of free water.

NaDC, instead, showed a characteristic profile since the variation in sound speed displays a sigmoidal-shape profile. In fact, there is not a clear breakpoint and a change in slopes between the two linear regions of sound speed corresponding to concentrations below and above CMC. This confirms that a a single point as CMC value cannot be calculated for NaDC .

The measured variation in sound speed below a surfactant concentration of 1 mM was less than 0.05 m/s, not allowing a reliable calculation of CMC for nonionic surfactants (CMC <1 mM for PEG8-L and PEG8-S) (Figure S10).

**Figure 6:**
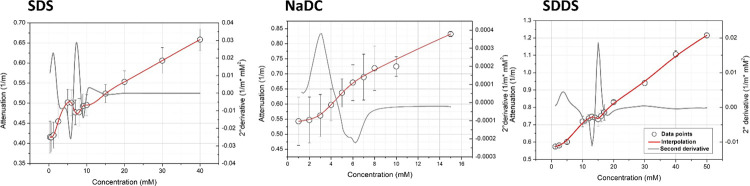
Attenuation *vs* concentration plots for anionic (SDS, NaDC, SDDS) surfactants.

Figure 6 shows the attenuation profiles for the analyzed anionic surfactants. As for sound speed, attenuation generally increases with surfactant concentrations and has a characteristic behavior in the proximity of CMC. As for sound speed and the other measured parameters, the attenuation profiles were similar for SDS and SDDS, showing two linear segments, which are referred to the concentration at which surfactants as unimers or micelles are predominant. In the proximity of CMC, a deflection in the increase of attenuation occurs, highlighting a minimum that could be identified as the CMC value. This deflection can be ascribed to an increase in the heterogeneity of the sample, occurring at concentrations close to CMC, and related to the appearance of micelles in bulk dispersion, thereby reflecting the dynamic evolution of the system. It can be also underlined that, as for sound speed, the slopes are higher in the unimeric state than those in the micellar state for both surfactants. This difference is much more pronounced for SDS, as already observed for the sound speed parameter.

CMC is an intrinsic value, reflecting the chemical–physical properties of surfactants, which can be determined for all pure amphiphiles or their mixture of known composition. The calculation of CMC is crucial since the physical properties of surfactants change with concentration, exhibiting a sharp discontinuity close to CMC. Despite the pivotal importance of this parameter, there are still some discrepancies in the literature regarding the calculated values also for commercial surfactants. Obviously, some differences are expected in the calculated values from various techniques, being measuring different chemical–physical properties related to the surface absorption or aggregation behavior of the amphiphile. However, many of such differences can be derived from the weakness of the used technique, the presence of impurities, or data analysis.^36^ In the last context, it is fundamental to develop such methods of analysis that are not dependent on the operator (as the straight-line method), thereby not affecting the result. Here, it is presented the possible use of the maximum value from the second derivative of raw data for calculating the CMC. from This approach can be applied to all used techniques, therefore enabling a more direct comparison among them. The CMC values obtained by the use of the second derivative (Table 1) were then compared with those obtained by the segmental linear regression (Table S1), which is an operator-free routine method employed for the calculation of CMC. There is a complete agreement between the CMC values calculated by the segmental linear regression and the maximum value of the second derivative, underlining the efficacy of the proposed method to calculate the CMC values in a reliable manner (Table 1).

**Table 1:** CMC Values Calculated by the Second Derivative of Raw Data for All Surfactants According to the Different Techniques Used

CMC (mM)						
	tensiometry	conductimetry	densimetry	fluorescence (pyrene)	sound speed	attenuation
SDS	6.53 ± 1.12	8.40 ± 1.14	8.84 ± 0.14	9.18 ± 0.76	8.58 ± 0.22	9.00 ± 0.83
NaDC	2.32 ± 0.61	7.08 ± 0.99	6.26 ± 0.69	8.20 ± 0.36	5.82 ± 1.36	6.19 ± 1.31
	4.40 ± 0.54					
SDDS	14.33 ± 0.45	14.25 ± 1.11	16.02 ± 0.49	16.38 ± 2.35	14.47 ± 0.54	15.04 ± 0.74
PEG8-L	0.10 ± 0.08	a	a	0.23 ± 0.02	a	a
PEG8-S	0.04 ± 0.02	a	a	0.06 ± 0.03	a	a
	0.26 ± 0.02			0.47 ± 0.03		

a No CMC values can be calculated from conductimetry, fluorescence, and HR-US data for PEG8-L and PEG8-S surfactants.

Only tensiometry and fluorescence spectroscopy can be employed for both the analyzed nonionic and anionic surfactants to determine CMC since the other techniques were restricted to anionic surfactants. As known, nonionic surfactants do not consistently affect the conductivity of solutions. For density and ultrasound measurements, the limitations of the technique were found for testing surfactant solutions of concentrations below 1 mM. At these low concentrations, the contribution to density, sound speed, or attenuation is not detected as accurate as required for the reliable determination of CMC. Therefore, these techniques (densimetry and ultrasound spectroscopy) are not suitable for the calculation of surfactants with a CMC lower than or around 1 mM, especially for most of the nonionic surfactants. The very small contribution in density or sound speed of surfactants at low concentrations is also reported in the literature.^37^

No marked differences have been found among the calculated CMC values using different techniques; however, the lowest values were calculated from tensiometry. The lowest values from tensiometry can be explained by the fact that the air–water surface can be saturated at a concentration below which surfactant micelles form, also due to the absorbance of more hydrophobic contaminants.^16^

On the other side, the highest CMC values for all surfactants were calculated by fluorescence spectroscopy using pyrene as a probe. Despite, sometimes, the fluorescence method using pyrene as a probe has been proposed to be versatile and precise, particularly useful for the determination of low CMC values as for amphiphilic copolymers,^14,38^ there is still an unequivocal procedure to obtain the CMC values from the experimental data. Indeed, for all surfactants, the plot made from the ratio I/III of vibrionic peaks of pyrene *vs* concentrations provides a sigmoidal curve, from which the CMC values were calculated using different approaches, sometimes empirical, as the intersection of straight lines fitting the experimental data or the fitting with a Boltzmann-type function (Figure S11).^15^ A thorough determination of CMC using pyrene should take into account several physical properties influencing the fluorescence emission of this probe in surfactant solutions. Fluorescence emission is, indeed, affected by the partition equilibrium between bulk water and micelles, the localization of pyrene inside or onto the surface of micelles, the possible quenching due to excimer formation, and the remaining interaction of pyrene with the surfactant as unimers.^39^ According to Zana and co-worker, the CMC values can be determined by fluorescence spectroscopy using pyrene as a probe by two different approaches, depending on the CMC value. For surfactants with a CMC >1 mM (generally ionic surfactants), the CMC values can be approximated to the intercept between the two lines fitting the rapidly decreasing portion and the nearly plateau portion at high concentrations in the pyrene I/III intensity *vs* surfactant concentration plot. On the other side, for surfactants with CMC <1 mM (generally nonionic surfactants), the CMC values can be approximated to the inflection point of the pyrene I/III intensity *vs* surfactant concentration plot as calculated by the Boltzmann regression.^40^ The CMC values obtained by the second derivative of fluorescence raw data are closer and comparable to those obtained by segmental linear regression (Table S1) with respect to those from the Boltzmann regression (Table S2). Thus, the second derivative approach is more suitable for the calculation of CMC for surfactants with a higher CMC (as ionic surfactants). Specifically, the Boltzmann equation provides the CMC values close to the real ones for pure nonionic surfactants, which display well-sigmoidal-shape plots. In the case of impurities, as for PEG8-S surfactants, the plot displays two decays and the Boltzmann regression provides only one averaged value (Table S2).

High-resolution ultrasonic spectroscopy is a powerful technique to study the colloidal behavior of nanosystems of different nature,^41^ but it has been employed in a very limited number of studies to calculate CMC.^37,42,43^ In these studies, the CMC values were determined by the intersection of the two straight lines fitting sound speed raw data, which are dependent on the aggregation state of the amphiphile. The variation in the other ultrasound parameters, such as attenuation, as a function of surfactant concentrations has been never practically considered for the determination of CMC, and no references are available in the literature about data treatment. Indeed, fluctuations in the ultrasound attenuation have been only theoretically postulated but never exploited in experimental studies to determine the CMC values.^44^

All of the analyses were performed on the commercial surfactants (anionic and nonionic) used without any further purification. This allows the comparison among all techniques, in relation to the proposed approach of the second derivative of raw data to calculate CMC, in a more realistic scenario than using amphiphiles with the highest grade of purity, after a further purification process by the experimenter. Indeed, the analyzed anionic surfactants (SDS, NaDC, and SDDS) have a purity higher than 98% (as declared by the manufacturer) and the calculated CMC values according to the “second derivative” approach are in the range of, or at least comparable to, the values reported on the label of the product (7–10 mM for SDS, 2–6 mM for NaDC, and 14.6 mM for SDDS) and reported in the literature for the compounds with a similar grade of purity^23,27,45,46^ (i.e., the theoretical CMC for pure SDS at 25 °C in water is 8.3 mM^47^). On the other side, the analyzed nonionic surfactants, *i.d.* PEG esters (PEG8-L and PEG8-S), have presumably a purity lower than that of the analyzed ionic surfactants, as suggested by ESI mass spectrometry and DSC analysis (Supporting Figures S1–S8). For these surfactants, the purity, as well as the expected CMC value, is not declared by the manufacturer. Indeed, these commercial surfactants are composed of a mixture of PEG esters, of which the declared amphiphile is only the prevalent one. According to this, any comparison of the CMC values from the literature for this class of nonionic compounds is far to be straightforward.

## Conclusions

The CMC values of surfactants can be reliably calculated using the maximum value of the second derivative from raw data collected by all analyzed techniques (tensiometry, conductimetry, densimetry, fluorescence spectroscopy, and high-resolution ultrasound spectroscopy) in an operator-free manner. Among all tested surfactants, not all techniques were effective in the calculation of CMC due to some limitations of the techniques themselves and the nature of the surfactants. CMC can be determined for all analyzed surfactants (both anionic and nonionic) only using tensiometry and fluorescence spectroscopy. On the other side, conductivity, densimetry, and ultrasound spectroscopy were effective only for anionic surfactants, which display higher CMC values (above 1 mM). As regards ultrasonic spectroscopy, both ultrasound parameters (sound speed and attenuation) can be successfully employed for the determination of CMC. Particularly, in this work, data for attenuation of ultrasounds were proposed for the calculation of CMC of surfactants, providing comparable results with the other techniques.

## Supporting Information Available

ESI mass spectra of all surfactants; DSC traces for all surfactants; conductivity *vs* concentration plot for the anionic surfactants; sound speed *vs* concentration plot for the PEG8-L surfactant; CMC values calculated by the segmental linear regression method for all surfactants according to the different techniques used and the CMC values calculated by the Boltzmann nonlinear fitting of fluorescence raw data; and fluorescence intensity (peak I/III) *vs* concentration plots for all surfactants (PDF)
