# Supplementary material for: Surfactant Self-Assembling and Critical Micelle Concentration: One Approach Fits All?
Source: Langmuir. 2020 May 6;36(21):5745–53. doi: 10.1021/acs.langmuir.0c00420 (PMC8007100; doi:10.1021/acs.langmuir.0c00420)
Supplement: Supplementary file 1 — la0c00420_si_001.pdf [file la0c00420_si_001.pdf]

Supporting information

Surfactants self-assembling and critical micelles concentration: one approach fits to all?

Diego Romano Perinelli[a], Marco Cespi[a], Nicola Lorusso[a], Giovanni Filippo Palmieri[a], Giulia Bonacucina[a*], Paolo Blasi[a†]

[a]School of Pharmacy, University of Camerino, 62032 Camerino (Italy);

Correspondence: giulia.bonacucina@unicam.it

Number of pages: 12

Number of figures: 11

Number of tables: 2

Electrospray ionization (ESI) Mass analysis

Each surfactant was dissolved in methanol and analysed by direct injection in an electron spray mass ionization (ESI) apparatus (HP 1100 150 LC/MSD, Agilent) equipped with a single quadrupole detector. SDS, NaDC and SDDS were analysed in the negative mode. PEG8-L and PEG8-S were analysed in the negative mode and positive mode. Fragmentor voltage is 30 V.

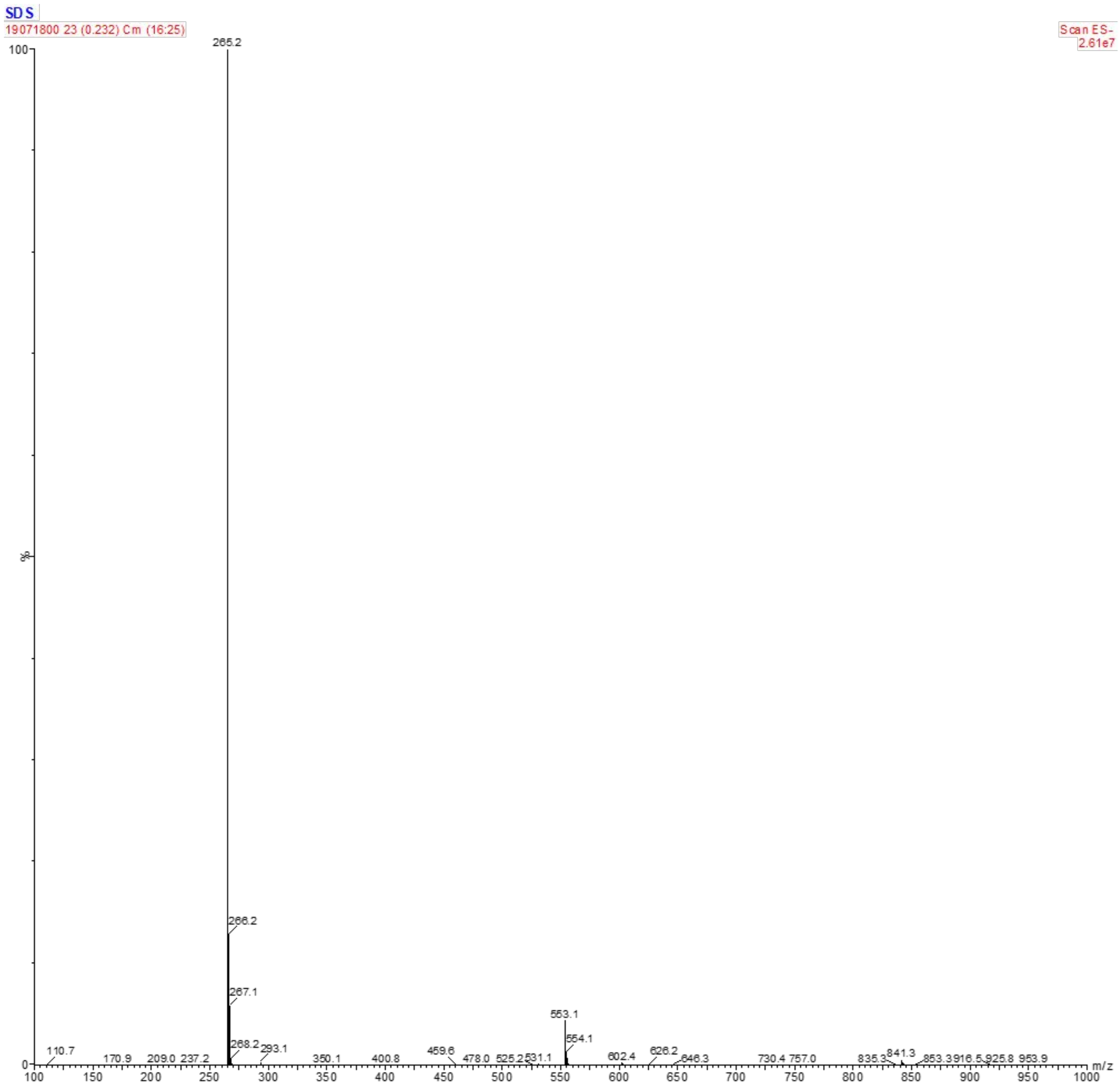

Figure S1 ESI mass spectrum (negative mode) of SDS

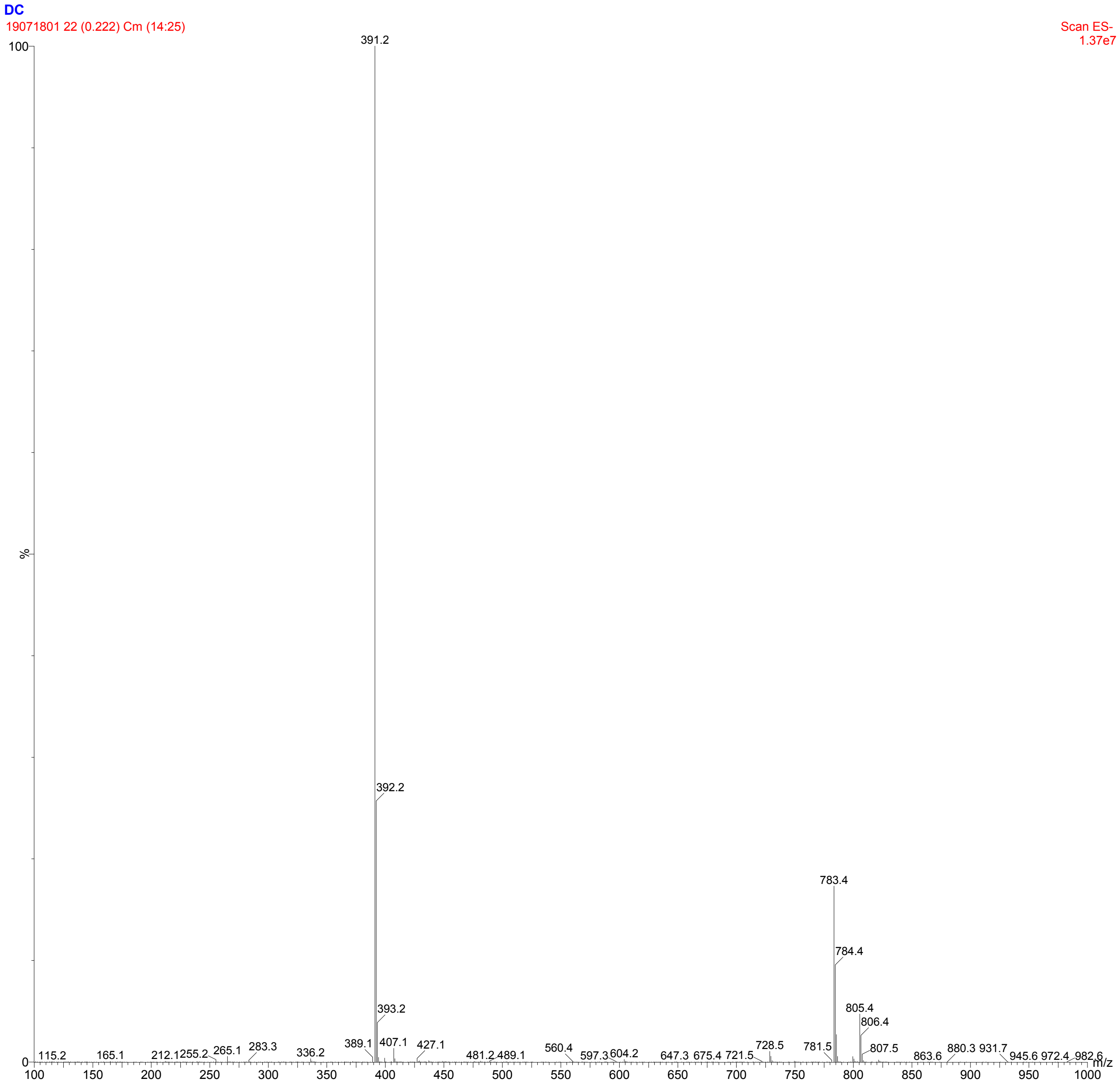

Figure S2 ESI mass spectrum (negative mode) of NaDC

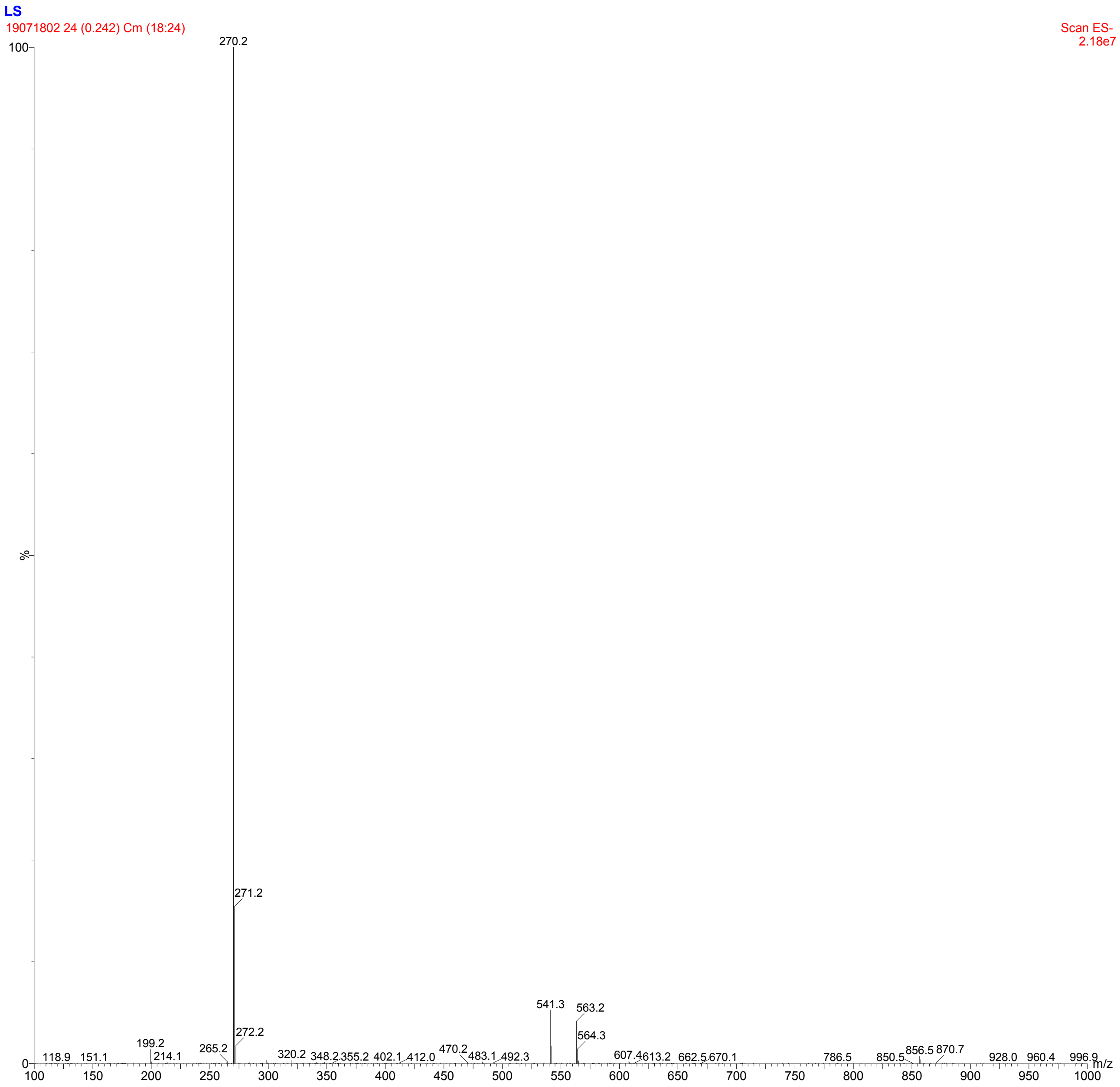

Figure S3 ESI mass spectrum (negative mode) of SDDS

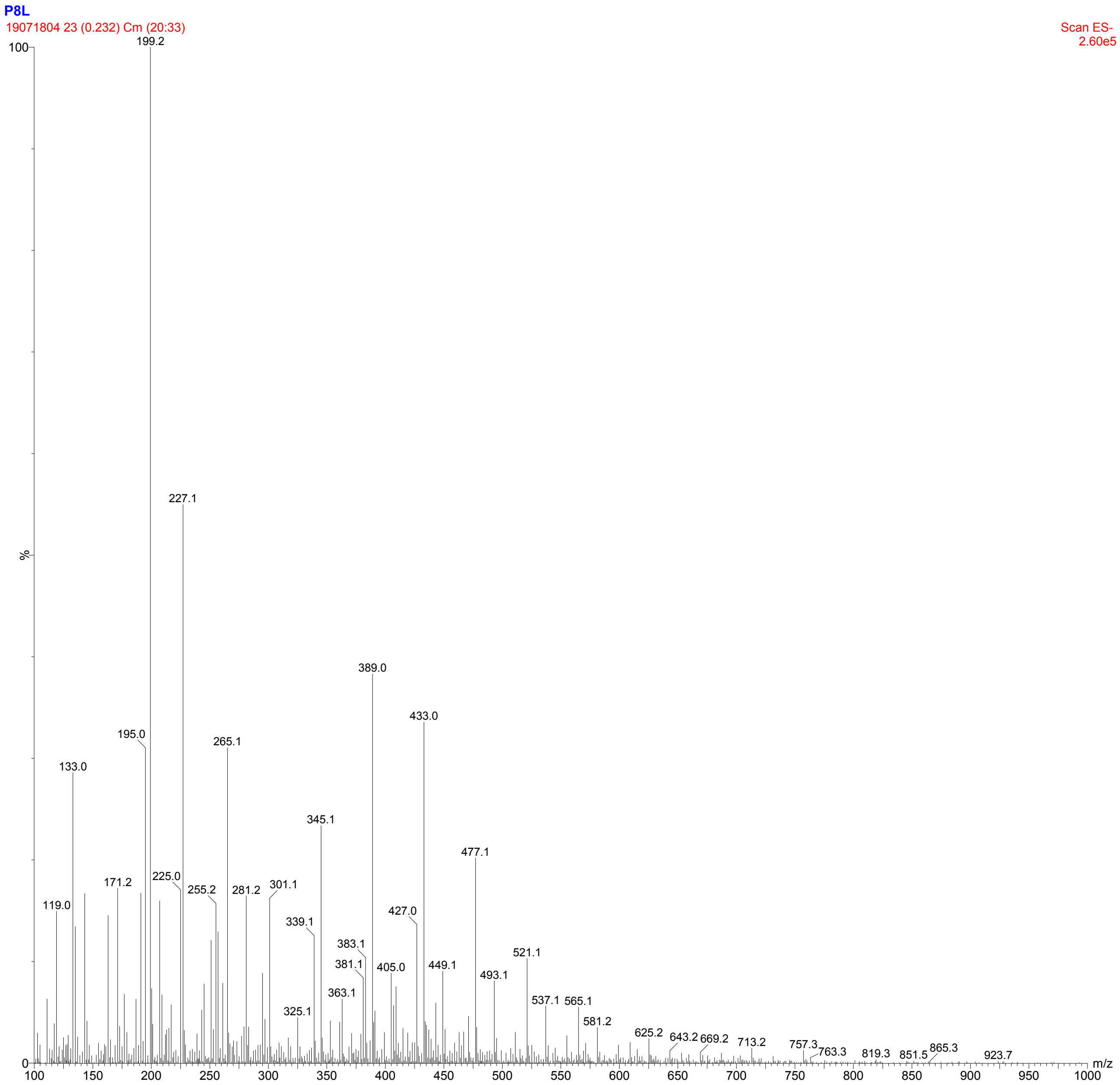

Figure S4 ESI mass spectrum (negative mode) of PEG8-L

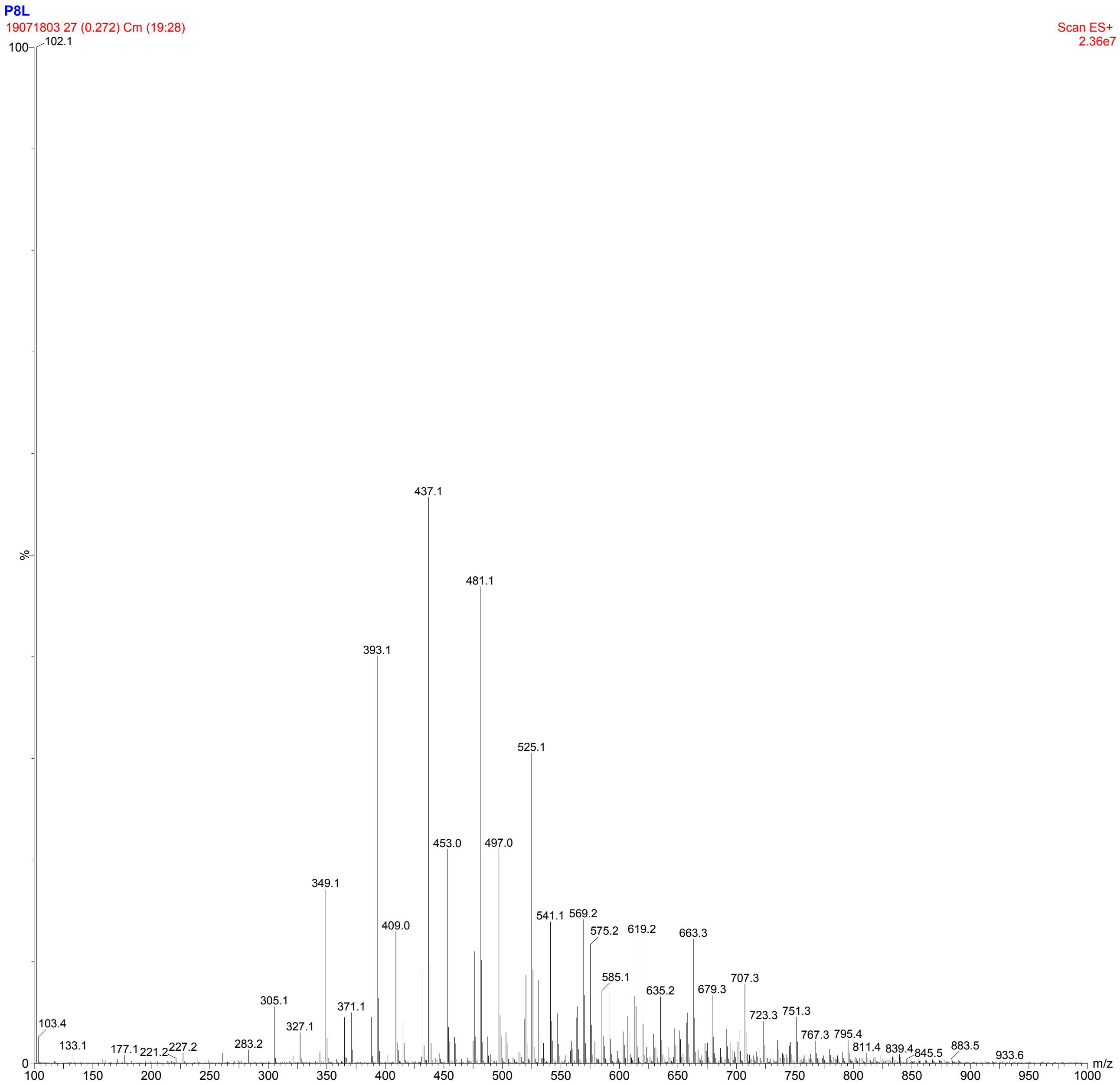

Figure S5 ESI mass spectrum (positive mode) of PEG8-L

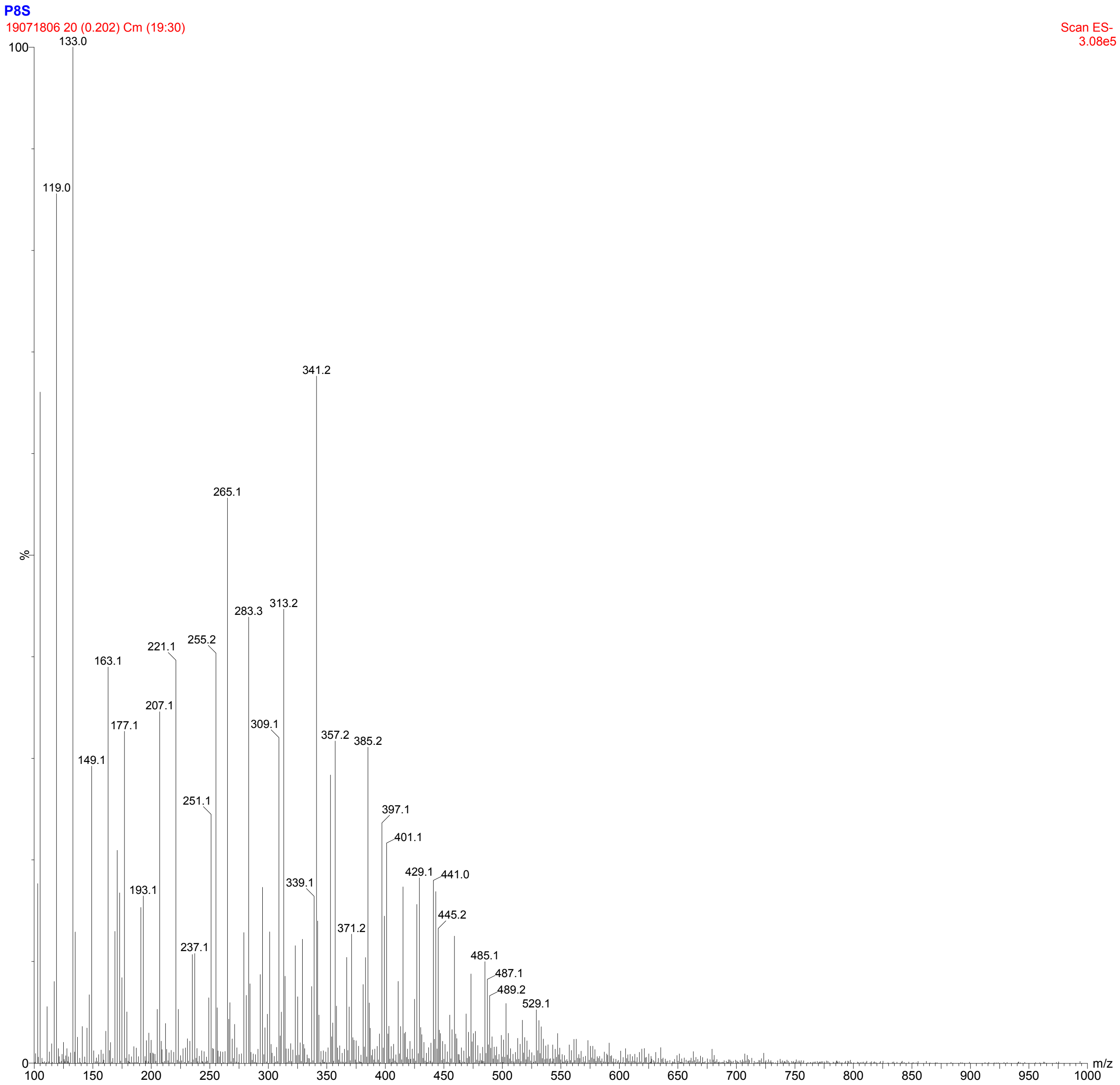

Figure S6 ESI mass spectrum (negative mode) of PEG8-S

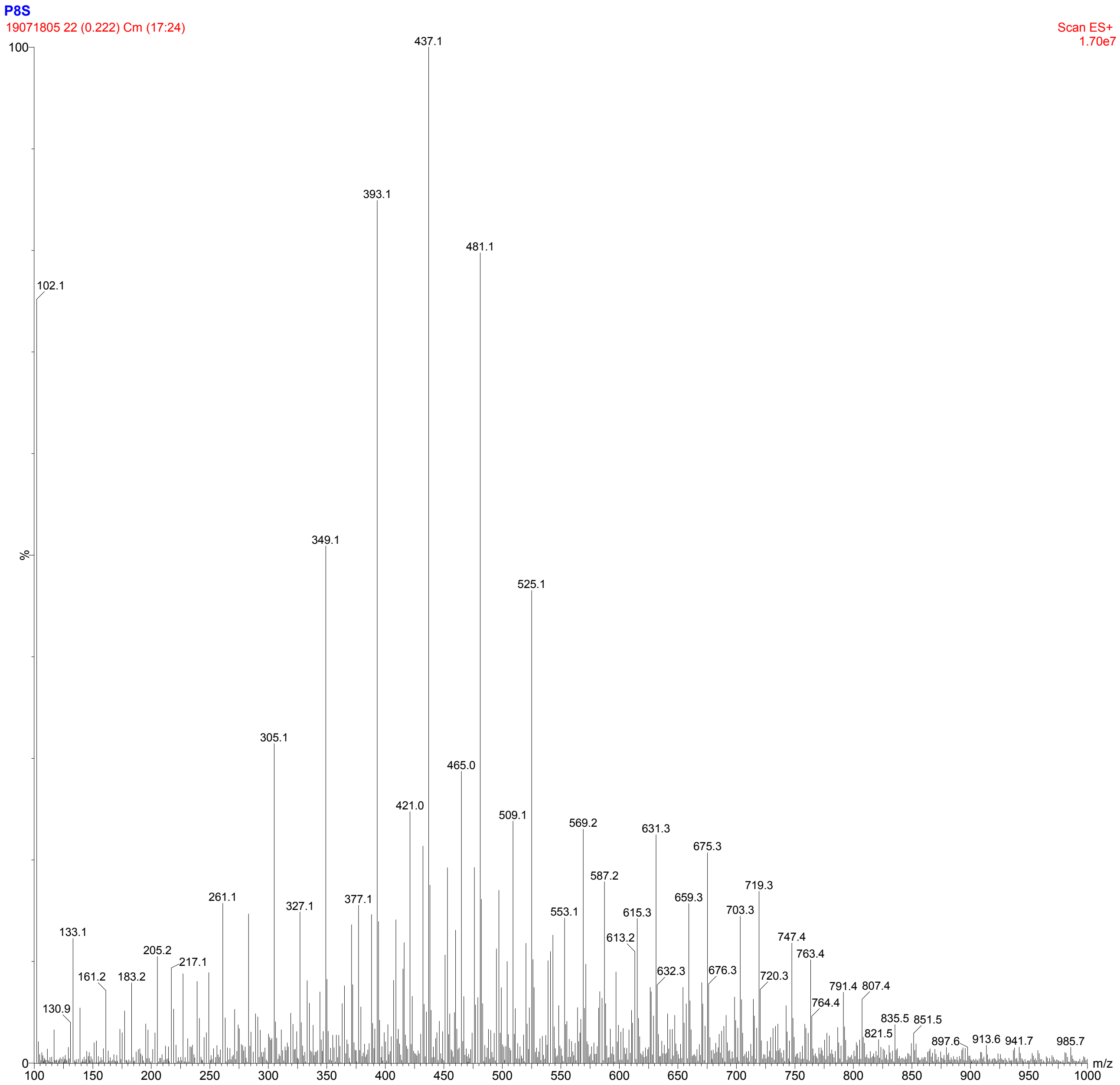

Figure S7 ESI mass spectrum (positive mode) of PEG8-S

Differential scanning calorimetry (DSC)

DSC thermograms were recorded using a DSC 8500 (PerkinElmer, Norwalk, USA), equipped with an intracooler (Intracooler 2, PerkinElmer, Norwalk, USA) in an inert nitrogen atmosphere. A small amount of the samples was placed in closed aluminum pans and analyzed by heating at 10 °C/min from 0 °C to 250 °C for SDS, NaDC and SDDS and from -50 °C to 100 °C for PEG8-L and PEG8-S surfactants.

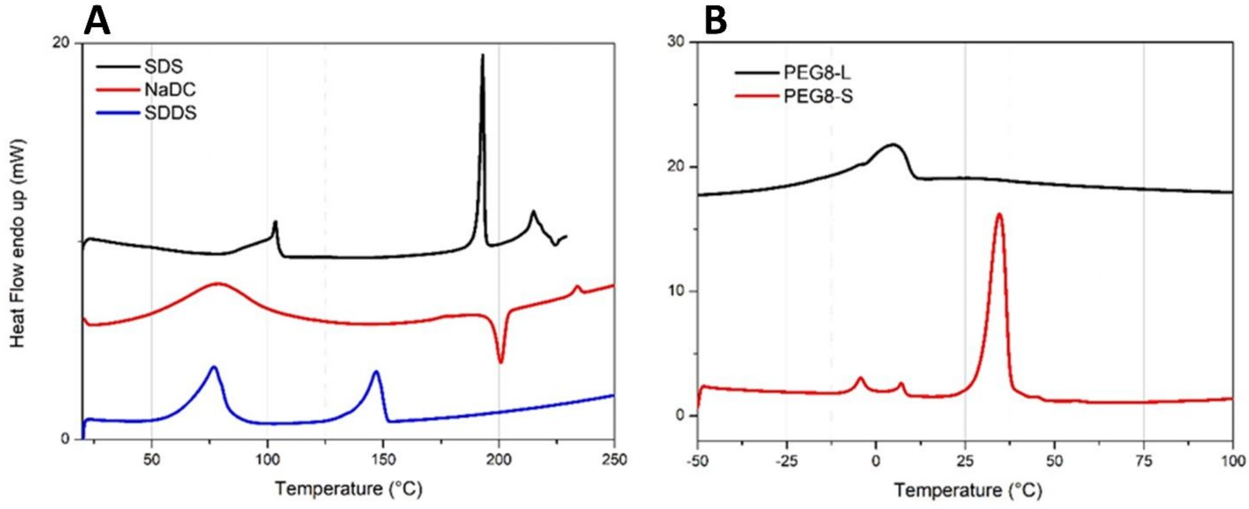

Figure S8 DSC traces for anionic surfactants (SDS, NaDC and SDDS; A) and non-ionic surfactants (PEG8-L and PEG-8S; B)

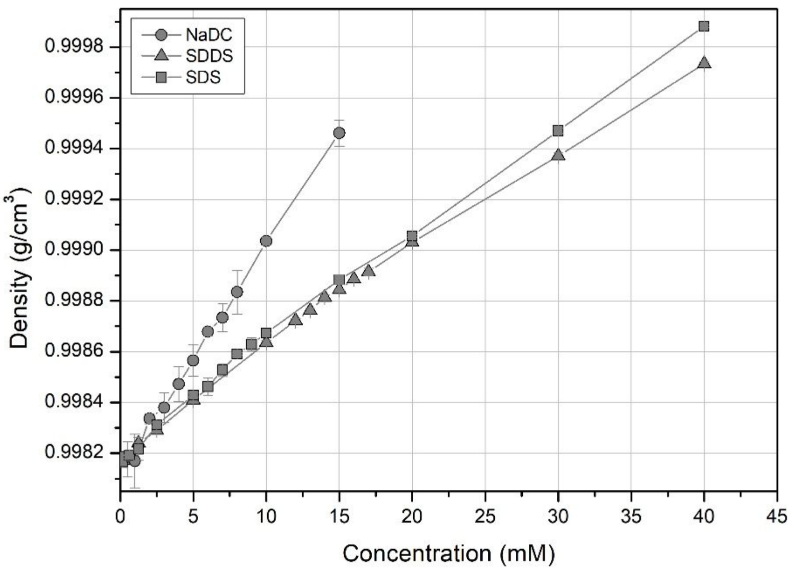

Figure S9 Conductivity *vs* concentration plot for the anionic surfactants (SDS, NaDC and SDDS)

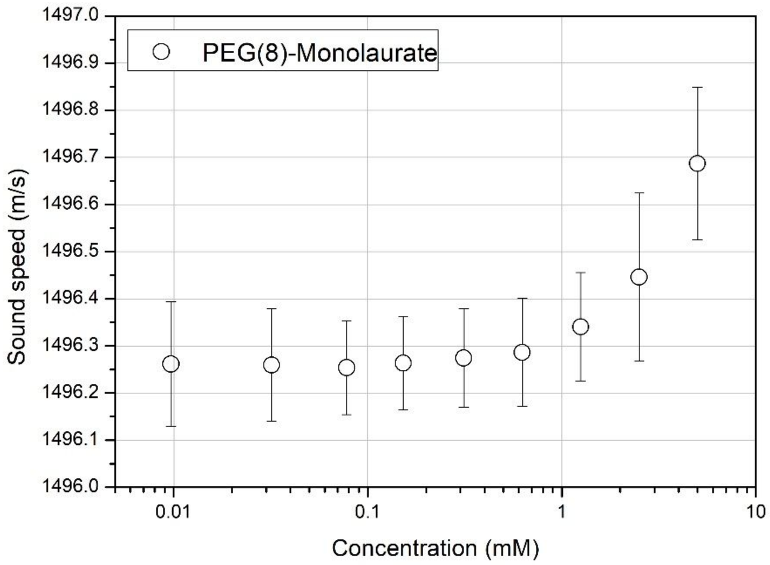

Figure S10 Sound speed *vs* concentration plot for PEG8-monolaurate surfactant

Table S1 CMC values calculated through the segmental linear regression method for all surfactants according the different techniques used

CMC (mM) Segmental linear regression						
	Tensiometry	Conductimetry	Densimetry	Fluorescence (pyrene)	Sound speed	Attenuation
SDS	6.20 ± 0.39 (0.980)	7.79 ± 0.27 (0.999)	8.30 ± 1.15 (0.999)	10.45 ± 1.92 (0.964)	8.64 ± 0.17 (0.997)	**
NaDC	2.14 ± 0.20 (0.778)	9.68 ± 2.83 (0.999)	5.98 ± 1.60 (0.991)	7.50 ± 0.01 (0.898)	7.28 ± 0.83 (0.997)	**
SDDS	16.70 ± 0.15 (0.992)	13.87 ± 0.33 (0.998)	14.18 ± 0.75 (0.999)	14.13 ± 8.74 (0.957)	14.32 ± 0.34 (0.998)	**
PEG8-L	0.057 ±0.028 (0.886)	*	*	0.102 ± 0.023 (0.971)	*	**
PEG8-S	0.046 ±0.022 (0.964)	*	*	0.362 ± 0.071 (0.968)	*	**

*No CMC values can be calculated from conductimetry, fluorescence and HR-US data for PEG8-L and PEG8-S surfactants

** Attenuation data cannot be fitted by the segmental linear regression method

The number into the brackets is the R^2 value calculated on the mean curve from three independent measurements

Table S2 CMC values calculated through the Boltzmann non-linear fitting of fluorescence raw data

	Fluorescence (pyrene)	R^2
SDS	4.88 ± 0.61	0.952
NaDC	4.90 ± 1.20	0.972
SDDS	5.48 ± 0.73	0.980
PEG8-L	0.036 ±0.004	0.983
PEG8-S	0.122 ±0.019	0.987

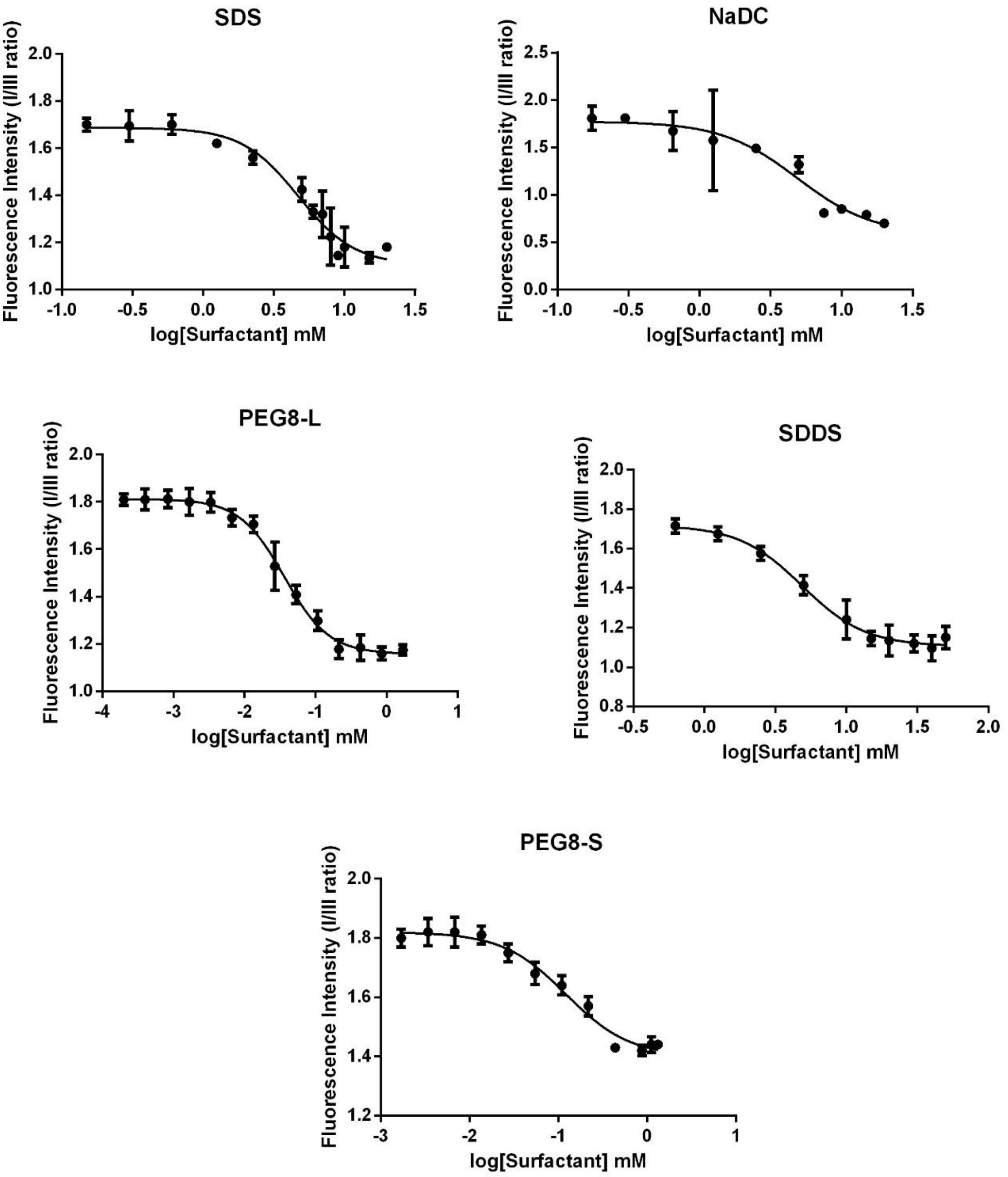

Figure S11 Fluorescence intensity (Peak I/III) *vs* concentration plots for ionic (SDS, NaDC, SDDS) and non ionic (PEG8-L and PEG8-S) surfactants. The line is the Boltzmann non-linear fitting of raw data.
